# The Effects of Emotion Regulation Treatment on Disruptive Behavior Problems in Children: A Randomized Controlled Trial

**DOI:** 10.1007/s10802-022-00903-7

**Published:** 2022-02-08

**Authors:** Urdur Njardvik, Hronn Smaradottir, Lars-Göran Öst

**Affiliations:** 1 Department of Psychology, University of Iceland, Reykjavik, Iceland; 2 Department of Psychology, Stockholm University, Stockholm, Sweden; 3 Bergen Center for Brain Plasticity, Haukeland University Hospital, Bergen, Norway

**Keywords:** Disruptive behavior problems, Externalizing disorders, Children, Cognitive behavioral treatment, Randomized controlled trial

## Abstract

Disruptive behavior problems are a frequent reason for children’s referrals to psychological services and can have negative effects on social and academic functioning. Most treatment programs involve parents as recipients and implementation of intervention programs in school is low. Deficits in emotion regulation have recently been implicated in the development of disruptive behavior disorders, making child directed early intervention programs focusing on increasing emotion regulation skills feasible. The purpose of this study was to assess the effects of Tuning Your Temper, a brief cognitive behavioral program for children with disruptive behavior problems, in a randomized controlled trial. A total of 125 children with disruptive behavior problems at school, aged 6–11 years old were randomized to either intervention or wait-list control condition. Treatment was conducted at school. Assessments included teacher and parent ratings on the Strengths and Difficulties Questionnaire (SDQ) and the Disruptive Behavior Rating Scale (DBRS) pre- and post-treatment and at 6-month follow-up. Results showed a significant reduction in behavior problems for the treatment condition on both measures and effects were maintained at 6-month follow-up. Results were more robust for teacher ratings, with medium to large effect sizes. Tuning Your Temper appears to be a promising early intervention program for children with disruptive behavior problems at school.

Disruptive behavior problems are one of the most common reasons for referral to psychological services for children in elementary school and are associated with long-term negative outcome (Buckley, 2009; Kassing et al., 2019; Kimonis et al., 2014). Children who display behavior problems at school often have difficulty meeting academic demands and struggle with social relationships with their peers (Clark et al., 2002; Daunic et al., 2012). They are also at an increased risk of developing emotional problems, as children with externalizing disorders are frequently diagnosed with depression and anxiety (Jarrett & Ollendick, 2008; Wolff & Ollendick, 2006). The first years in elementary school seem to be a period of particular vulnerability, when the onset of Oppositional Defiant Disorder (ODD) is prominent and many comorbid problems seem to develop (Frick & Matlasz, 2018; Kessler et al., 2005, 2007; Kim-Cohen et al., 2005; Merikangas et al., 2009; Tremblay et al., 2013). It has also been shown that externalizing behavior problems become increasingly resistant to change with age (Bernazzani et al., 2001; Deković & Stoltz, 2015). Furthermore, disruptive behavior disorders in childhood can have serious detrimental sequalae such as a higher risk of unemployment, criminality and mortality in adulthood (Scott et al., 2017). The development of effective treatments for children with disruptive behavior problems is therefore important, both for the individuals’ quality of life and for societal and economic reasons.

Several school-wide behavioral support systems and curriculum based programs have been developed to reduce children’s disruptive behavior problems at school. These include the school-wide Positive Behavior Interventions and Supports (PBIS), which has been shown to be an effective prevention and intervention for children with emotional and behavioral difficulties and in providing a school environment where evidence-based social behavioral support strategies are implemented (Lewis et al., 2010; Sadler & Sugai, 2009). Several universally delivered curriculum-based classroom interventions have also been shown to be effective prevention programs for children’s behavioral, social and emotional difficulties such as the Incredible Years Dinosaur curriculum (Webster-Stratton & Reid, 2003) and the Tools for Getting Along curriculum, which has been demonstrated to maintain treatment effects for two years (Daunic et al., 2019; Smith et al., 2014).

There is also considerable evidence supporting the use of specific cognitive behavioral treatment (CBT) for children with disruptive behavior problems. This includes both children diagnosed with externalizing disorders such as ODD, Conduct Disorder (CD) and Attention Deficit Hyperactivity Disorder (ADHD) and children with more broadly defined disruptive behavior problems. For externalizing disorders, the evidence shows that CBT is effective in routine clinical care with large effect sizes (Riise et al., 2021). Several CBT based programs have also been shown to effectively reduce disruptive behavior problems in children who have been referred to services or are considered at risk (Lochman et al., 2011), thus supporting the use of CBT both for children diagnosed with behavior disorders and those presenting with milder disruptive behavior problems.

Several studies have explored which treatment components are most effective in treating disruptive behavior problems, what treatment setting is preferable, and which target behaviors seem to be most responsive to intervention. The treatment components identified as most effective include positive parenting skills, emotion awareness, affective education, arousal reduction, problem solving, goal setting, and perspective taking (Gansle, 2005; Lochman et al., 2011; Sukhodolsky et al., 2004). Treatment format and setting do not seem to significantly affect treatment outcome among children with externalizing disorders (Riise et al., 2021), making group treatment delivered at school an attractive option as that would provide easy access to services for most children. In fact, several studies have demonstrated the effectiveness of treatment delivered at school. Meta-analyses of school-based studies have found that cognitive strategies targeting the reduction of aggressive behavior or anger are effective when used in the school setting and provide lasting results (Gansle, 2005; Robinson et al., 1999).

As explained above, several school-wide and curriculum based universally administered programs have been developed and adopted successfully in many school districts. However, for some children and some schools, a more individualized intervention approach may be feasible to reduce the frequency and/or severity of disruptive behaviors. Many of such interventions consist of multiple sessions and although effective abbreviated versions have been developed for some of these programs, they are still quite lengthy. For example, the Coping Power Program, which in the full version consists of 34 child sessions and 16 parent sessions, has been found to be effective in an abbreviated version consisting of 24 child sessions and 10 parent sessions (Lochman et al., 2014). Many school-based programs, including the Coping Power Program and the Aggression Replacement Training (ART), are also designed for children in the 5th or 6th grade (Goldstein et al., 1998; Lochman et al., 2014), an age which has been identified as a critical period and an optimal time to increase adaptive behavior (Jurecska et al., 2011). However, studies show that the presence of aggression and oppositional behavior in the 1–3rd grade predicts adolescent delinquency (Buckley, 2009; Dishion et al., 2014). Early intervention is therefore warranted and while there is support for many such behavioral programs focusing on parents (i.e. Incredible Years, Parent–Child Interaction Therapy [PCIT] and Positive Parenting Program [Triple P], the development of more varied interventions for early disruptive behavior problems has been called for (Comer et al., 2013).

The majority of treatment interventions for externalizing disorders involve parents either as the sole recipient or together with the child (Riise et al., 2021) and behavioral parent training programs have been shown to be effective in reducing children’s behavioral problems and increasing compliance (Burkey et al., 2018; Kaminski & Claussen, 2017). Most parenting programs focus on increasing the children’s compliance through behavioral modification strategies. This approach conceptualizes the children’s behavior problems as a performance deficit which can be resolved by changing environmental contingencies. In the recent literature, however, the relation between emotion regulation and disruptive behavior problems has received increased attention. In the DSM-5 (APA, 2013), symptoms of ODD have, for example, been divided into categories separating irritability and mood related symptoms from defiant behavior and vindictiveness and the ICD-11 includes a specifier for ODD with chronic angry and irritable mood (WHO, 2019). While the diagnostic criteria itself did not change, this subcategorization has increased the focus on the emotional aspect of the disorder (APA, 2013; Burke & Romano-Verthelyi, 2018; Frick & Matlasz, 2018; Ollendick et al., 2018) and several studies have supported a link between emotion regulation and the development of disruptive behavior problems in children (e.g. Cole et al., 2003; Stringaris et al., 2010; Mitchison et al., 2020). Emerging disruptive behavior problems in children may thus be related to deficits in emotion regulation which would constitute a skill deficit as opposed to a performance deficit. Early interventions focusing on increasing emotion regulation skills may therefore be effective in reducing disruptive behavior problems in children and could potentially be delivered at school with minimal parental involvement.

The purpose of this study was to assess the effects of Tuning Your Temper, a brief cognitive behavioral program, on disruptive behavioral problems in children. The effects of the program were assessed in a randomized controlled trial conducted in a school setting. The program is based on CBT strategies such as arousal reduction, problem solving and perspective taking which have been shown to be efficacious in reducing externalizing behavior problems and aggressive behavior in children (e.g. Lochman et al., 2011; Sukhodolsky et al., 2004). In light of studies supporting the use of these treatment components, it was expected that children receiving Tuning Your Temper would show greater reduction in disruptive behavior problems than children in the wait-list control condition, and that these effects would be retained at follow-up assessment six months post treatment.

## Method

### Participants

Participants were referred to the study by the schools’ psychologists. To be included in the study children had to previously have been referred to psychological services due to behavioral problems at school (i.e. verbal/physical aggression, noncompliance, temper outbursts or frequent conflicts with peers) and be Icelandic speaking. Since a referral to psychological services was an inclusion criterion, in some instances school psychologists referred children from their waiting lists to the study. Exclusion criteria were IQ < 70 and a diagnosis of an autism spectrum disorder but as some children were on the waiting lists for services at their school, this information was not available for all children. The study was conducted in two waves. In a few instances in wave 1, children received a diagnosis of autism spectrum disorder (ASD) or were measured as having an IQ < 70 after the treatment had started. For ethical reasons these children were allowed to complete the treatment but their data were not used in the final analysis. In wave 2 the inclusion criteria were made clearer to avoid this issue.

Seven schools in the greater metropolitan area of Reykjavik participated in the study. A total of 144 children in 2nd-5th grade were referred to the study but parents of two children declined participation, so 142 children were randomized to either intervention or wait-list control condition. The randomization sequence was created using Excel 2007 with a 1:1 allocation using random block sizes, performed by a person who was not involved in the study in any way. Families and teachers did not remain blind to condition status after random assignment as some children attended treatment sessions at school and others had to wait. The participants were originally randomized in two different samples, using slightly different age criteria. Based on reviewers’ suggestion, these samples were collapsed into one study consisting of two waves. In wave 1, 80 children from 2nd-5th grade in five elementary schools were randomized to either treatment or waitlist control condition. In wave 2, 62 children from 2nd-4th grade were randomized to the same conditions. The participants in wave 2 also came from five elementary schools, three of the schools had also participated in wave 1, but two schools were new to the study.

**Fig. 1:**
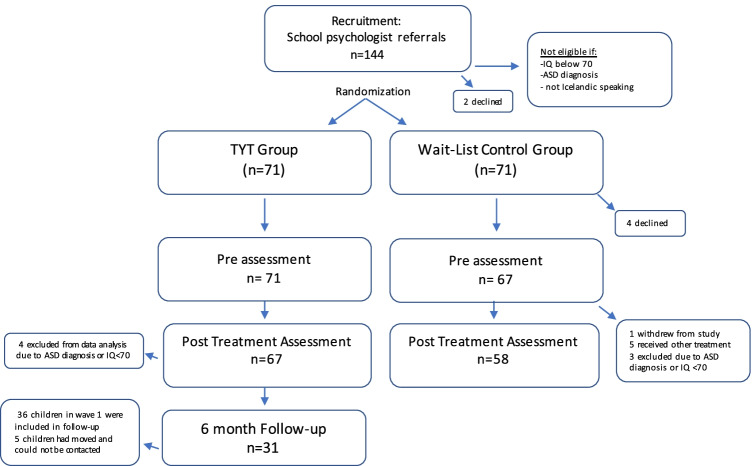
Flow chart of participation

In the control condition, parents of four children withdrew from the study prior to completing the pre-treatment assessment and one child was withdrawn from the study after the assessment. Five children dropped out as they received other treatment and three children were removed from analysis as they received a diagnosis of ASD or had an IQ < 70. Additionally, four children in the treatment condition received a diagnosis of ASD or had an IQ < 70 and were therefore removed from the analysis, leaving a total sample of 125 children, 105 boys (84%) and 20 girls (16%), 67 in the treatment condition and 58 in the control condition. The children were 6–11 years old, with a mean age of 8.60 (SD 1.10). In wave 1, follow-up assessment was conducted for the treatment condition at six months post treatment. As five of the 36 children in the treatment condition (14%) had moved away and could no longer be contacted through the schools, follow-up data were obtained from 31 participants (86%) (see Fig. 1 and Table 1).

**Table 1:** Baseline demographic information

	**Total**	**TYT program**	**Control condition**
**Child characteristics**	**(N = 125)**	**(n = 67)**	**(n = 58)**
*Gender*			
* Boys*	105 (84.0%)	55 (82.1%)	50 (86.2%)
* Girls*	20 (16.0%)	12 (17.9%)	8 (13.8%)
*Age M (SD)*	8.60 (1.10)	8.65 (1.10)	8.61 (1.11)
*% on medication*	17.60%	17.90%	17.20%
**Parent characteristics**			
Respondent			
*mother*	87.30%	83.90%	91.10%
*father*	7.60%	8.10%	7.10%
*mother & father together*	5.10%	8.00%	1.80%
Age			
*20–29*	11.90%	6.50%	17.90%
*30–39*	65.30%	72.60%	57.10%
*40–49*	22.00%	21.00%	23.20%
*50–59*	0.80%	0%	1.80%
Marital status			
*Married/cohabiting*	58.30%	65.00%	50.90%
*Single parent*	41.70%	35.00%	49.10%
Educational status			
*Compulsory education*	56.40%	55.70%	57.20%
*Secondary/Vocational*	24.80%	26.20%	23.20%
*University degree*	18.80%	18.00%	19.60%
**Teacher characteristics**			
Gender			
*male*	0.80%	1.50%	0%
*female*	98.80%	98.50%	100%
Age			
*20–29*	0.80%	0%	1.70%
*30–39*	32.00%	32.80%	31.00%
*40–49*	48.00%	52.20%	43.10%
*50–59*	15.20%	11.90%	19.00%
*60* +	4.00%	3.00%	5.20%
Time knowing the child			
< *6 months*	16.80%	17.90%	15.50%
*6–12 months*	24.00%	23.80%	24.20%
*1–2 years*	33.60%	29.80%	37.90%
> *2 years*	25.60%	28.40%	22.40%
Number of children in class M (SD)	20.5 (SD 5.0)	19.9 (SD 3.5)	20.5 (SD 5.0)

## Measures

### Demographic Questions

The study included standard demographic questions, i.e. child’s age, gender and medication status; parents’ age, gender, education and marital status; teachers’ age, number of children in their class and how long they had known the child. Questions about race and family income were not included as it is neither customary nor culturally accepted to request such information in Icelandic studies.

### The Strengths and Difficulties Questionnaire (SDQ)

The SDQ is a brief screening instrument designed to assess behavioral and emotional difficulties in children. The instrument consists of 25 questions rated on a three-point Likert scale. Results are divided into five subscales: emotional symptoms, conduct problems, hyperactivity/inattention, peer relationship problems, and prosocial behavior (Goodman, 1997). In the current study, the parent and teacher reports were used. Results are reported from the conduct problems subscale which consists of items intended to screen for ODD and conduct disorder (i.e. “*often has temper tantrums or hot tempers”* and “*often fights with other children or bullies them”*). The SDQ has been translated and standardized in Icelandic and its psychometric properties have been found to be acceptable with good concurrent and predictive validity as well as acceptable internal consistency (α = 0.71) (Skarphedinsson & Magnusson, 2008). Similar results were found in the present sample (α = 0.79).

### Disruptive Behavior Rating Scale (DBRS)

The ODD section of the DBRS was used in this study and was completed by both parents and teachers. It is designed to assess symptoms of ODD and the items are based on the DSM-IV diagnostic criteria (i.e. “*argues with adults*”, “*loses temper*”, and “*is angry or resentful*”). The scale consists of eight items rated on a four-point Likert scale, with a clinical cut-off score of 12. The scale has good psychometric properties and the Icelandic version has good interrater reliability and internal consistency (α = 0.93) (Barkley, 2013; Mitchison et al., 2020). Similar results were found in the present study (α = 0.91). The DBRS has also been found to have good convergent/divergent and discriminative validity (Friedman-Weieneth et al., 2009).

### Treatment Intervention

*Tuning Your Temper* is a brief cognitive behavioral program for 6–12 year old children with behavioral difficulties. It is designed to be delivered in group format and comprises a therapist manual, children’s work book, homework assignments and weekly letters to parents or guardians. The program consists of six weekly 60 min sessions focusing on three factors: 1) *Knowledge and understanding*: Children learn to recognize their emotional reactions and become aware of the situations where their weaknesses are most likely to cause difficulties. 2) *Behavioral control*: Children learn and practice new ways to control their emotional reactions and express their emotions and wishes in a more adaptive manner. 3) *Changed way of thinking*: Children overcome their potential hostile attribution bias, assess their environment in a more detailed manner and use a greater number of environmental cues before choosing to react.

In session 1, the children work on assignments where their temper is explored in terms of physical, cognitive, behavioral and environmental factors. Through pictorial assignments the children explore and measure aspects of their temper, i.e. by filling in missing parts of pictures; circling pictures and words which represent the way they experience their difficult temper (what they feel, think and how they behave); circling items from a list of different situations that trigger their temper (i.e. *I get upset when someone cheats in a game*); and coloring a measurement tape to indicate how strong their reaction is in different situations. From these pictorial assignments a pattern will emerge which the therapist uses to help the child see in which types of situations they seem to be most vulnerable. In session 2, various ways of controlling your temper are discussed and arousal reduction techniques are introduced. These include a simple breathing exercise and the use of a “stress-ball” which the children make during the session. Goal setting is introduced and the children set up a plan targeting specific problem situations. In session 3, the therapists introduce problem solving techniques, i.e. how to define a problem, generate potential solutions, assess their quality and pick a good one. What defines a good solution is discussed and the children work on assignments related to actual problems from their own environment. In session 4, problem solving training continues and cognitive restructuring is introduced. The focus here is on thoughts which overestimate the negative aspects of a situation (i.e. only seeing one side of the problem, seeing things as black-or-white, believing you know what someone else is thinking without asking them). In session 5, cognitive restructuring continues and exercises aimed at increasing the child’s self-esteem are introduced (i.e. *Find five things that you are good at or make you a good person. Then draw your hand and write one item on each finger to help you remember)*. Finally, in session 6, the focus is on summarizing the materials and how the children should maintain the skills they learned during the treatment.

During each session the children worked individually and collectively on various assignments assisted by their therapists. At the end of each session the children were given a small toy as a reward, a home-work assignment and a letter to take home to their parents or guardians. The letter informed the parents about each week’s topic as well as explaining the homework assignments. In both waves of this study the sessions were conducted at school during school hours. There were 6–8 children and two therapists per group. Attendance rate was high, 86.6% of the treatment condition participants attended all six sessions and the remaining 13.4% missed one session.

### Therapists

A total of six therapists were involved in the treatment. All had completed a master’s degree in clinical psychology, completed clinical practicum training and four of them were licensed psychologists. All received weekly supervision by the first author. Randomly selected treatment sessions (40% of sessions) were also observed and the therapists received feedback during supervision sessions to encourage adherence to the manual, but treatment fidelity was not scored in a formal way.

### Procedure

Institutional review board approval was obtained from the National Bioethics Committee in Iceland (VSNb2009120010/03.7). Seven schools participated in the study, five in each wave. School psychologists were asked to refer children who had originally been referred to psychological services due to behavior problems and/or frequent conflicts with peers. Once the school personnel had explained the study and obtained written informed consent from parents/legal guardians, the children were randomized to either intervention or wait-list control condition. The study was also explained to the children by their teachers. Assessment instruments were administered to parents and teachers pre- and post-intervention. In wave 1, follow-up assessment was conducted six months post treatment. As it was not deemed ethical to withhold treatment from children in the control condition for longer than two months, follow-up data were not gathered from them.

### Data Analysis

Data were analyzed using SPSS v.27. Potential differences between conditions on demographic variables were assessed using chi-square and independent *t-*tests. Main effects for treatment gains were assessed using repeated measures ANOVA with condition as a between subjects factor, time as a within subjects factor and wave as a covariant. When a significant interaction between condition and time was found this was followed by an independent *t*-test to test if the change scores for the two conditions were different. When a significant interaction between condition and time was not found, but a significant effect of time was found, this was followed with an independent *t*-test between the treatment conditions at post-treatment. This was done to test if it was the treatment or both conditions that were responsible for the effect of time. As only the treatment condition in wave 1 participated in the follow-up assessment (n = 31) a paired samples *t*-test was conducted to assess whether treatment gains were retained. Finally, within-group effect sizes were calculated by using Cohen’s *d*. Missing values were first analysed using Little’s MCAR test, which was non-significant *(χ*^*2*^* (108, N* = *125)* = 118.6, *p* = 0.229). Then, missing values were imputed using multiple imputation analysis (*m* = 5) and a pooling procedure with the standard error estimates combined for posttest analyses. Thus, an intent-to-treat analysis is used in this study.

### Nesting Effects

As the results could potentially be affected by school culture, we analyzed potential threat to independence of the data within schools. For each of the outcome variables, Intra Class Correlation coefficients (ICCs) were calculated for school membership. The ICCs were 0.032 (95% CI: –0.100–0.185), and 0.112 (95% CI: 0.001–0.350) for conduct problems (SDQ) at pre and post intervention, respectively, and –0.093 (95% CI:.–0.134–0.055), and 0.001 (95% CI: –0.129 –0.152) for the DBRS. As all ICC’s were below 0.30, levels of dependence between the members of the same cluster are considered to be low.

Similarly, potential threat of nesting within therapists was also tested using ICCs. There were six therapists working in teams of two so ICCs were calculated for the three therapist pairs. ICCs for post treatment data on the DBRS (ICC for teacher ratings = 0.003 (95% CI –0.122–0.162); ICC for parent ratings = 0.136 (95% CI –0.009–0.282)) and the conduct problems subscale of the SDQ (ICC for teacher ratings = 0.028 (95% CI –0.106–0.163); ICC for parent ratings = 0.076 (95% CI –0.015–0.148)) were all below 0.30, thus indicating that threat to independence of data due to nesting within therapists was low.

## Results

No significant differences were found between the two conditions on any of the demographic variables (see Table 1) or clinical variables at pre-treatment (Table 2).

**Table 2:** Means, SD and effect sizes for teacher and parent ratings

					**Within-group ES (*d*)**	
**Measures**	**Condition**	**Pre treatment**	**Post treatment**	**Follow-up**	**Post**	**Follow-up**
***Teacher ratings***						
SDQ conduct	Treatment	70.64 (16.65)	64.04 (15.05)**	62.03 (14.37)*	0.419	0.545
	Control	68.76 (13.97)	68.62 (13.95)		0.010	
DBRS	Treatment	12.90 (6.30)	9.94 (5.55)*	9.23 (5.94)*	0.502	0.599
	Control	13.10 (6.53)	12.44 (6.53)		0.102	
***Parent ratings***						
SDQ conduct	Treatment	62.50 (14.05)	58.45 (13.74)	56.76 (9.40)*	0.294	0.454
	Control	63.04 (13.65)	61.10 (13.17)		0.146	
DBRS	Treatment	9.62 (5.53)	7.96 (4.73)	5.66 (3.24)**	0.325	0.812
	Control	8.66 (4.98)	7.88 (4.65)		0.164	

*SDQ* Strengths and Difficulties Questionnaire, *DBRS* Disruptive Behavior Rating Scale

^*^p < 0.05; **p < 0.01

### Teacher ratings

The results are presented in Table 2. On the SDQ conduct subscale there was a significant main effect of time *F(1,123)* = 12.08,* p* < 0.01; but not of condition *F(1,123)* = 130.32,* p* = 0.56. There was a significant interaction between time and condition *F(1,123)* = 10.80, *p* < 0.01 and between time and wave *F(1,123)* = 7.49, *p* < 0.01. An independent *t*-test showed that the treatment condition had a significantly larger change score than the control condition *t(123)* = 3.18, *p* < 0.01 and that in the treatment condition wave 2 had a significantly larger change score than wave 1 (*t(65)* = 2.00, *p* < 0.05). On the DBRS the teacher ratings revealed a similar pattern with a significant main effect of time *F(1,123)* = 21.21, *p* < 0001; but not of condition *F(1,123)* = 2.50, *p* = 0.12; and a significant interaction between time and condition *F(1,123)* = 8.49, *p* < 0.01 but not between time and wave *F(1,123)* = 3.50, *p* = 0.06. Here too the treatment condition improved significantly more than the control condition, *t(123)* = 2.40, *p* < 0.05*.* At 6-month follow-up these treatment gains were maintained for the intervention condition (wave 1 only) as the difference between pre-treatment and follow-up assessment was significant on the DBRS, *t(30)* = 2.64, *p* < 0.05 as well as on the SDQ Conduct Problems, *t(30)* = 2.53, *p* < 0.05.

### Parent Ratings

On the SDQ conduct subscale, there was a significant main effect of time *F(1,123)* = 16.56, *p* < 0.01; but not of condition *F(1,123)* = 0.54,* p* = 0.46, or interaction between time and condition *F(1,123)* = 2.47,* p* = 0.12 or between time and wave *F(1,123)* = 0.004,* p* = 0.95. As there was a main effect of time, a follow-up test was conducted to see if the treatment condition or both conditions were responsible for the effect of time. No significant differences were found between the conditions’ post-treatment scores *t(123)* = 1.09, *p* = *0*.27. At follow-up there was a significant difference between pre-treatment and follow-up means *t(30)* = 2.26, *p* < 0.05 for the treatment condition. On the DBRS the parent ratings revealed a significant main effect of time *F(1,123)* = 18.86, *p* < 0.001 but not of condition *F(1,123)* = 0.51,* p* = 0.47, or interaction between time and condition *F(1,123)* = 3.29,* p* = 0.07; or between time and wave *F(1,123)* = 0.28,* p* = 0.91. There was no significant difference between the conditions’ post-treatment scores *t(123)* = 0.95, *p* = *0*.92. However, the pre- to follow-up change score was significant for the treatment condition in wave 1, *t(30)* = 3.39, *p* < 0.01.

## Discussion

The purpose of this study was to assess the effects of a brief CBT group intervention for children with disruptive behavior problems. The program, Tuning Your Temper, was tested in a randomized controlled trial, conducted in a school setting. The results showed that, in only six sessions, Tuning Your Temper can significantly reduce disruptive behavior problems in children in the earlier grades of elementary school, according to both parent and teacher ratings.

The results revealed a statistically significant reduction in teacher rated conduct problems and ODD symptoms in the treatment condition and results were maintained at 6-month follow-up. We did not find a statistically significant reduction in parent rated behavior problems except at follow-up. The follow-up data were, however, only available for a part of the treatment condition and not for the control condition which should be taken into account when interpreting these results. Effect sizes were small for parent ratings and medium for teacher ratings at post-treatment, but were medium to large at follow-up (see Table 2).

Teacher ratings of both conduct problems and ODD symptoms for the treatment condition indicated a meaningful change as the mean scores went from being above clinical cut-off at pre-treatment to below clinical cut-off at post-treatment. Follow-up data were only available for a portion for the treatment condition but showed that the mean scores were maintained below clinical cut-off scores at 6-month follow-up. Parent ratings pre-treatment were below clinical cut-off, which can be explained by the fact that the inclusion criterion was a referral to psychological services by school personnel and some of the children may therefore not have displayed behavior problems at home. The fact that findings were more robust with larger effect sizes for teacher ratings may also be attributable to the fact that in the treatment sessions, there was a strong focus on problems with peer interaction and dealing with difficult demands from adults. This may lead to greater improvements seen in the school environment as opposed to the home.

The results were in congruence with previous research on the effectiveness of using problem solving, arousal reduction and cognitive strategies to reduce aggression and externalizing behavior problems in children (e.g. Lochman et al., 2011; Sukhodolsky et al., 2004). In a majority of the schools participating in this study there was already a school-wide positive behavior support program in place, so the TYT intervention was an addition to a structured behavioral program and the children in the control condition were, thus, also receiving positive behavior support. The current findings therefore lend support to the use of specific skills training with children displaying behavioral difficulties in addition to the use of environmental contingencies.

The findings are also consistent with previous research showing that interventions delivered in school settings can be effective in reducing disruptive behavior problems (Gansle, 2005; Robinson et al., 1999). Furthermore, the current sample was fairly young, with a mean age of 8.6 years, which is encouraging as previous studies have shown that the onset of oppositional behaviors is most prominent in the first years of elementary school (Frick & Matlasz, 2018; Kessler et al., 2005, 2007; Kim-Cohen et al., 2005; Merikangas et al., 2009; Tremblay et al., 2013) and disruptive behavior problems become more resistant to change with age (Bernazzani et al., 2001; Deković & Stoltz, 2015).

In the present study the treatment intervention approaches the child’s problem as a difficulty with controlling their temper. Several previous studies have focused on anger in treatment of disruptive behavior problems, as it relates to aggression, hostility and oppositional behavior which are frequent reasons for referrals (Sukhodolsky et al., 2004). During the development of this intervention, the initial version of Tuning Your Temper used the words “angry” or “anger” in the children’s assignments. We found, however, that many children did not relate to being angry when their behavioral difficulties were discussed, which resulted in the wording being changed from anger management to tuning the temper. The children seemed more receptive of these terms which is perhaps consistent with research connecting emotion dysregulation and oppositional behavior in children, as evidence shows that emotion dysregulation is a risk factor for the development of ODD symptoms (Calkins et al., 2019; Liu et al., 2019; Martel et al., 2012; Stringaris et al., 2010). Irritability is also a factor which is frequently implicated in disruptive behavior problems and studies show that irritability is related to aggressive reactive behaviors when children feel frustrated (Brotman et al., 2017; Stringaris & Goodman, 2009). Approaching the children’s problem as an issue with controlling their temper as opposed to discussing anger or defiance in this treatment intervention, may therefore have made the children more receptive to the study material, which in turn may have contributed to its effect.

The main research question being tested in this study was whether young children in the early grades of elementary school would show improvements in behavior after a very brief CBT intervention delivered at school with minimal parental participation. In this program, parents only received weekly information letters about the week’s topic and homework assignments and the child only attended six weekly sessions during school hours. The program therefore did not require the parents to commit to attending any sessions or having to transport their children anywhere. The results indicate that Tuning Your Temper, or a similar child directed school based treatment, could potentially serve as an early intervention strategy to reduce disruptive behavior problems in young children.

The findings of this study were strengthened by the fact that we conducted a randomized controlled trial and were able conduct the study within a school setting. Another strength is the inclusion of both parent and teacher ratings of the children’s behavior. There were also several limitations to the study that need to be considered when interpreting the results. First, the inclusion criteria were not based on a cut-off score or a clinical diagnosis, but rather that the children had been referred to psychological services due to behavioral problems. While this probably provided a realistic sample of children presenting with behavior problems in school, some of them may have had mild problems with little room for improvement. Second, follow-up assessment was not available for the control condition as it was deemed unethical to withhold treatment from those children for 6 months. Third, the outcome measures did not include an assessment of emotion regulation, which makes it difficult to determine if the intervention led to changes in emotion regulation skills. Fourth, in wave 1, children from the school psychologists’ waiting lists were included in the study which resulted in some of them being excluded from the analysis after receiving a diagnosis of intellectual deficits or ASD during the treatment phase. In wave 2 this was remedied which led to lower attrition. Fifth, as the treatment was delivered at school, teachers were not blind to which children were in the treatment condition and this may have affected the results. Finally, although 40% of randomly selected sessions were observed in order to encourage adherence to the treatment manual, treatment fidelity was not scored in a formal way. Future studies should include a more defined clinical sample, a larger sample in follow-up assessment and an assessment of the children’s knowledge of the intervention’s content.

In conclusion, the results of this study show that Tuning Your Temper, a brief CBT intervention for children, was effective in reducing disruptive behavior problems as rated by both teachers and parents. The results were more robust for teacher ratings than for parent ratings, with medium to large effect sizes, and showed that CBT can effectively reduce disruptive behavior problems in young children in just six sessions with minimal parental involvement. Although more research is needed to further establish the efficacy of this treatment program, Tuning Your Temper appears to be a promising early intervention program for young children displaying disruptive behavior problems at school.

## Data Availability

Data is available upon request.

## References

[CR1] American Psychiatric Association (2013). Diagnostic and Statistical Manual of Mental Disorders.

[CR2] Barkley, R. A. (2013). *Defiant children: A clinician's manual for assessment and parent training*: Guilford press.

[CR3] Bernazzani O, Côté C, Tremblay RE (2001). Early parent training to prevent disruptive behavior problems and delinquency in children. The Annals of the American Academy of Political and Social Science.

[CR4] Brotman MA, Kircanski K, Leibenluft E (2017). Irritability in children and adolescents. Annual Review of Clinical Psychology.

[CR5] Buckley JA (2009). Introduction to this special issue. Journal of Emotional and Behavioral Disorders.

[CR6] Burke JD, Romano-Verthelyi AM, Martel MM (2018). Oppositional defiant disorder. Developmental pathways to disruptive, impulse-control and conduct disorders.

[CR7] Burkey MD, Hosein M, Morton I, Purgato M, Adi A, Kurzrok M, Tol WA (2018). Psychosocial interventions for disruptive behaviour problems in children in low-and middle-income countries: A systematic review and meta-analysis. Journal of Child Psychology and Psychiatry.

[CR8] Calkins SD, Dollar JM, Wideman L (2019). Temperamental vulnerability to emotion dysregulation and risk for mental and physical health challenges. Development and Psychopathology.

[CR9] Clark C, Prior M, Kinsella G (2002). The relationship between executive function abilities, adaptive behaviour, and academic achievement in children with externalising behaviour problems. Journal of Child Psychology and Psychiatry.

[CR10] Cole PM, Teti LO, Zahn-Waxler C (2003). Mutual emotion regulation and the stability of conduct problems between preschool and early school age. Development and Psychopathology.

[CR11] Comer JS, Chow C, Chan PT, Cooper-Vince C, Wilson LA (2013). Psychosocial treatment efficacy for disruptive behavior problems in very young children: A meta-analytic examination. Journal of the American Academy of Child & Adolescent Psychiatry.

[CR12] Daunic AP, Smith SW, Garvan CW, Barber BR, Becker MK, Peters CD, Naranjo AH (2012). Reducing developmental risk for emotional/behavioral problems: A randomized controlled trial examining the Tools for Getting Along curriculum. Journal of School Psychology.

[CR13] Daunic AP, Smith SW, Aydin B, Barber B (2019). Lowering risk for significant behavior problems through cognitive-behavioral intervention: Effects of the Tools for getting along curriculum 2 Years following implementation. The Journal of Primary Prevention.

[CR14] Deković M, Stoltz S, Maric M, Prins PJM, Ollendick TH (2015). Moderators and mediators of treatments for youth who show externalizing problem behavior. Moderators and mediators of youth treatment outcomes.

[CR15] Dishion TJ, Brennan LM, Shaw DS, McEachern AD, Wilson MN, Jo B (2014). Prevention of problem behavior through annual family check-ups in early childhood: Intervention effects from home to early elementary school. Journal of Abnormal Child Psychology.

[CR16] Frick PJ, Matlasz TM, Martel MM (2018). Disruptive, impulse-control, and conduct disorders. Developmental pathways to disruptive, impulse-control and conduct disorders.

[CR17] Friedman-Weieneth JL, Doctoroff GL, Harvey EA, Goldstein LH (2009). The disruptive behavior rating scale–parent version (DBRS-PV) factor analytic structure and validity among young preschool children. Journal of Attention Disorders.

[CR18] Gansle KA (2005). The effectiveness of school-based anger interventions and programs: A meta-analysis. Journal of School Psychology.

[CR19] Goldstein, A. P., Glick, B., & Gibbs, J. C. (1998). *Aggression replacement training: A comprehensive intervention for aggressive youth* (Rev. ed.). Research Press.

[CR20] Goodman R (1997). The Strengths and Difficulties Questionnaire: A research note. Journal of Child Psychology and Psychiatry.

[CR21] Jarrett MA, Ollendick TH (2008). A conceptual review of the comorbidity of attention-deficit/hyperactivity disorder and anxiety: Implications for future research and practice. Clinical Psychology Review.

[CR22] Jurecska DE, Hamilton EB, Peterson MA (2011). Effectiveness of the Coping Power Program in middle-school children with disruptive behaviours and hyperactivity difficulties. Support for Learning.

[CR23] Kaminski JW, Claussen AH (2017). Evidence base update for psychosocial treatments for disruptive behaviors in children. Journal of Clinical Child & Adolescent Psychology.

[CR24] Kassing F, Godwin J, Lochman JE, Coie JD, Conduct Problems Prevention Research Group (2019). Using early childhood behavior problems to predict adult convictions. Journal of Abnormal Child Psychology.

[CR25] Kessler RC, Amminger GP, Aguilar-Gaxiola S, Alonso J, Lee S, Ustun TB (2007). Age of onset of mental disorders: a review of recent literature. Current Opinion in Psychiatry.

[CR26] Kessler RC, Berglund P, Demler O, Jin R, Merikangas KR, Walters EE (2005). Lifetime prevalence and age-of-onset distributions of DSM-IV disorders in the National Comorbidity Survey Replication. Archives of General Psychiatry.

[CR27] Kim-Cohen J, Arseneault L, Caspi A, Tomás MP, Taylor A, Moffitt TE (2005). Validity of DSM-IV conduct disorder in 4½–5-year-old children: A longitudinal epidemiological study. American Journal of Psychiatry.

[CR28] Kimonis E, Frick P, McMahon R, Mash EJ, Barkley RA (2014). Conduct and oppositional defiant disorders. Child psychopathology.

[CR29] Lewis TJ, Jones SE, Horner RH, Sugai G (2010). School-wide positive behavior support and students with emotional/behavioral disorders: Implications for prevention, identification and intervention. Exceptionality.

[CR30] Liu L, Chen W, Vitoratou S, Sun L, Yu X, Hagger-Johnson G, Wang Y (2019). Is emotional lability distinct from “angry/irritable mood”,“negative affect”, or other subdimensions of oppositional defiant disorder in children with ADHD?. Journal of Attention Disorders.

[CR31] Lochman JE, Baden RE, Boxmeyer CL, Powell NP, Qu L, Salekin KL, Windle M (2014). Does a booster intervention augment the preventive effects of an abbreviated version of the coping power program for aggressive children?. Journal of Abnormal Child Psychology.

[CR32] Lochman JE, Powell NP, Boxmeyer CL, Jimenez-Camargo L (2011). Cognitive-behavioral therapy for externalizing disorders in children and adolescents. Child and Adolescent Psychiatric Clinics.

[CR33] Martel MM, Gremillion ML, Roberts B (2012). Temperament and common disruptive behavior problems in preschool. Personality and Individual Differences.

[CR34] Merikangas KR, Nakamura EF, Kessler RC (2009). Epidemiology of mental disorders in children and adolescents. Dialogues in Clinical Neuroscience.

[CR35] Mitchison GM, Liber JM, Hannesdottir DK, Njardvik U (2020). Emotion dysregulation, ODD and conduct problems in a sample of five and six-year-old children. Child Psychiatry & Human Development.

[CR36] Ollendick TH, Booker JA, Ryan S, Greene RW (2018). Testing multiple conceptualizations of oppositional defiant disorder in youth. Journal of Clinical Child and Adolescent Psychology.

[CR37] Riise EN, Wergeland GJ, Njardvik U, Öst LG (2021). Cognitive Behavior Therapy for Externalizing Disorders in Children and Adolescents in Routine Clinical Care: A Systematic Review and Meta-analysis. Clinical Psychology Review.

[CR38] Robinson TR, Smith SW, Miller MD, Brownell MT (1999). Cognitive behavior modification of hyperactivity–impulsivity and aggression: A meta-analysis of school-based studies. Journal of Educational Psychology.

[CR39] Sadler C, Sugai G (2009). Effective behavior and instructional support: A district model for early identification and prevention of reading and behavior problems. Journal of Positive Behavior Interventions.

[CR40] Scott JG, Pedersen MG, Erskine HE, Bikic A, Demontis D, McGrath JJ, Dalsgaard S (2017). Mortality in individuals with disruptive behavior disorders diagnosed by specialist services–A nationwide cohort study. Psychiatry Research.

[CR41] Skarphedinsson G, Magnusson P (2008). Manual: Strengths and Difficulties Questionnaire.

[CR42] Smith SW, Daunic AP, Barber BR, Aydin B, Van Loan CL, Taylor GG (2014). Preventing risk for significant behavior problems through a cognitive-behavioral intervention: Effects of the Tools for Getting Along curriculum at one-year follow-up. The Journal of Primary Prevention.

[CR43] Stringaris A, Goodman R (2009). Three dimensions of oppositionality in youth. Journal of Child Psychology and Psychiatry.

[CR44] Stringaris A, Maughan B, Goodman R (2010). What's in a disruptive disorder? Temperamental antecedents of oppositional defiant disorder: Findings from the Avon longitudinal study. Journal of the American Academy of Child & Adolescent Psychiatry.

[CR45] Sukhodolsky DG, Kassinove H, Gorman BS (2004). Cognitive-behavioral therapy for anger in children and adolescents: A meta-analysis. Aggression and Violent Behavior.

[CR46] Tremblay M, Duchesne S, Vitaro F, Tremblay RE (2013). Developmental trajectories of oppositional behavior during elementary school and their risk factors. Journal of Educational and Developmental Psychology.

[CR47] Webster-Stratton C, Reid MJ (2003). Treating conduct problems and strengthening social and emotional competence in young children: The Dina Dinosaur treatment program. Journal of Emotional and Behavioral Disorders.

[CR48] Wolff JC, Ollendick TH (2006). The comorbidity of conduct problems and depression in childhood and adolescence. Clinical Child and Family Psychology Review.

[CR49] World Health Organization. (2019). *International Statistical Classification of Diseases and Related Health Problems* (11th ed.). https://icd.who.int/

